# Excitonic Structure and Primary Charge Separation in Purple Bacterial and Heliobacterial Reaction Centers

**DOI:** 10.3390/plants15182837

**Published:** 2026-09-16

**Authors:** Chenhui Wang, Yin Song

**Affiliations:** Beijing Key Laboratory of Intelligent Optoelectronic Sensing Technology and Instruments, School of Optics and Photonics, Beijing Institute of Technology, Beijing 100081, China

**Keywords:** charge separation, coherence, photosynthesis, excited-state dynamics, reaction centers

## Abstract

Photosynthetic reaction centers (RCs) are highly optimized pigment–protein complexes that drive the primary charge separation essential for solar energy conversion. Despite deep evolutionary divergence, many RCs share a conserved two-branch structural architecture, raising fundamental questions about how physical symmetry, excitonic interactions, and protein environments dictate electron-transfer directionality and efficiency. This review provides a comparative analysis of primary charge separation in two paradigmatic systems—the pseudo-symmetric purple bacterial reaction center (PbRC, Type II) and the homodimeric heliobacterial reaction center (HbRC, Type I). We first examine their structural and excitonic properties and discuss how protein-induced energetic asymmetry in PbRC favors electron transfer along a single active branch, whereas the symmetric HbRC supports charge separation through two equivalent branches, involving an A_0_-centered intermediate. We then highlight insights from ultrafast and multidimensional spectroscopy and first-principles calculations into charge-transfer intermediates, excitonic interactions, and coherent dynamics during the earliest stages of photochemistry. Finally, we discuss how evolutionary changes in cofactor identity, orientation, separation, and protein electrostatics may have shaped the distinct charge-separation mechanisms of Type I and Type II RCs. Together, these comparisons illustrate how photosynthetic RCs combine excitonic coupling, cofactor energetics, and protein-mediated electrostatic tuning to achieve efficient primary charge separation.

## 1. Introduction

Photosynthetic reaction centers (RCs) are highly evolved pigment–protein complexes that convert solar energy into chemical energy through a cascade of ultrafast excitation and electron-transfer processes [1,2,3]. Following light absorption and excitation-energy transfer from antenna pigments, RCs initiate the fundamental photochemical reaction of photosynthesis: primary charge separation, in which an electronically excited pigment state is converted into a spatially separated radical pair. Based on the identity of the terminal electron acceptor, photosynthetic RCs are broadly classified into two major families: Type I RCs, which transfer electrons to iron–sulfur clusters, and Type II RCs, which utilize quinone cofactors as electron acceptors [4]. Type I RCs include photosystem I (PSI) and heliobacterial reaction centers (HbRCs), whereas Type II RCs include photosystem II (PSII) and purple bacterial reaction centers (PbRCs) [4]. Despite their distinct cofactors and evolutionary divergence, many RCs share a conserved architecture consisting of two approximately symmetry-related cofactor branches surrounding a central pair of (bacterio)chlorophyll pigments, suggesting a common evolutionary origin [5]. The preservation of this two-branch arrangement raises a fundamental question: how do photosynthetic RCs achieve highly directional and efficient charge separation despite their apparent structural symmetry?

Primary charge separation is governed by the interplay between excitonic interactions, charge-transfer-state energetics, and protein-controlled electronic structures [5,6,7]. Light absorption initially generates electronically coupled excited states involving multiple pigments, with varying degrees of excitonic delocalization depending on pigment arrangement and electronic coupling strength. Subsequent charge separation proceeds through the formation of short-lived charge-transfer intermediates and stabilized radical pairs. The efficiency and directionality of this process are determined not only by pigment distances but also by site energies, electronic couplings, redox potentials, hydrogen-bonding interactions, electrostatic environments, and coupling between electronic states and nuclear motions. In many RCs, the initially formed excited states cannot be described as isolated pigment excitations but rather as delocalized or coupled exciton–charge-transfer states [8,9,10,11]. Ultrafast spectroscopy, particularly transient absorption, and two-dimensional electronic spectroscopy (2DES) have been widely applied to investigate excitonic and charge-separation dynamics in photosynthetic RCs [11,12,13,14,15,16,17,18,19,20,21,22,23,24,25,26]. It has been reported that excitonic delocalization and vibronic coupling can modulate the accessibility and mixing of charge-transfer states. However, whether experimentally observed coherence directly contributes to enhanced charge-separation efficiency remains an unresolved question [9,11,22,25].

This review examines primary charge separation in PbRC and HbRC as two contrasting realizations of a conserved reaction-center architecture. PbRC represents a pseudo-symmetric Type II RC in which protein-induced energetic asymmetry favors electron transfer along a single active branch, whereas HbRC represents a homodimeric Type I RC in which structurally equivalent branches participate in charge separation possibly involving an A_0_-centered intermediate. Together, these two systems provide complementary model platforms for elucidating how structural symmetry, cofactor energetics, and protein environments govern primary charge separation in Type II and Type I RCs, respectively [27,28]. In the following sections, we first summarize the structural and excitonic properties of PbRC and HbRC, then discuss their primary charge-separation pathways, and finally examine the possible roles of electronic and vibrational coherence in PbRC. Given the extensive literature on PbRC [11,22,23,27,29,30] and HbRC [2,4,24,31,32,33,34,35,36], as well as several reviews and books [27,37,38,39] covering theoretical and experimental studies of charge separation in photosynthetic RCs, this review focuses specifically on the relationships among structural organization, excitonic interactions, and primary charge-separation dynamics in PbRC and HbRC, with particular emphasis on how these factors govern their distinct charge-separation mechanisms. Through this comparative perspective, we aim to provide a coherent picture of the prevailing charge-separation mechanisms in these two systems while highlighting unresolved questions and continuing debate.

## 2. Charge Separation in PbRC

PbRC was identified through early spectroscopic studies of bacterial photosynthesis in 1952 and was first isolated as a photochemically active complex from *Rhodobacter sphaeroides* in 1968 [40]. In the 1970s, spectroscopic studies established that the primary donor is a bacteriochlorophyll dimer, bacteriopheophytin acts as a transient intermediate acceptor, and the quinones serve as the primary and secondary stable electron acceptors [40]. The later determination of high-resolution (<3 Å) X-ray crystal structures of the reaction centers from *Rhodopseudomonas viridis* and *R. sphaeroides* from 1984 to 1988 revealed the spatial organization of the electron donors and acceptors, providing a structural basis for quantitative analysis of the electron-transfer kinetics underlying primary charge separation [39]. In the following, we begin with the crystal structure of PbRC, followed by discussions of its excitonic structure and charge-separation mechanisms, with a particular emphasis on recent studies.

### 2.1. Structure of PbRC

**Figure 1:**
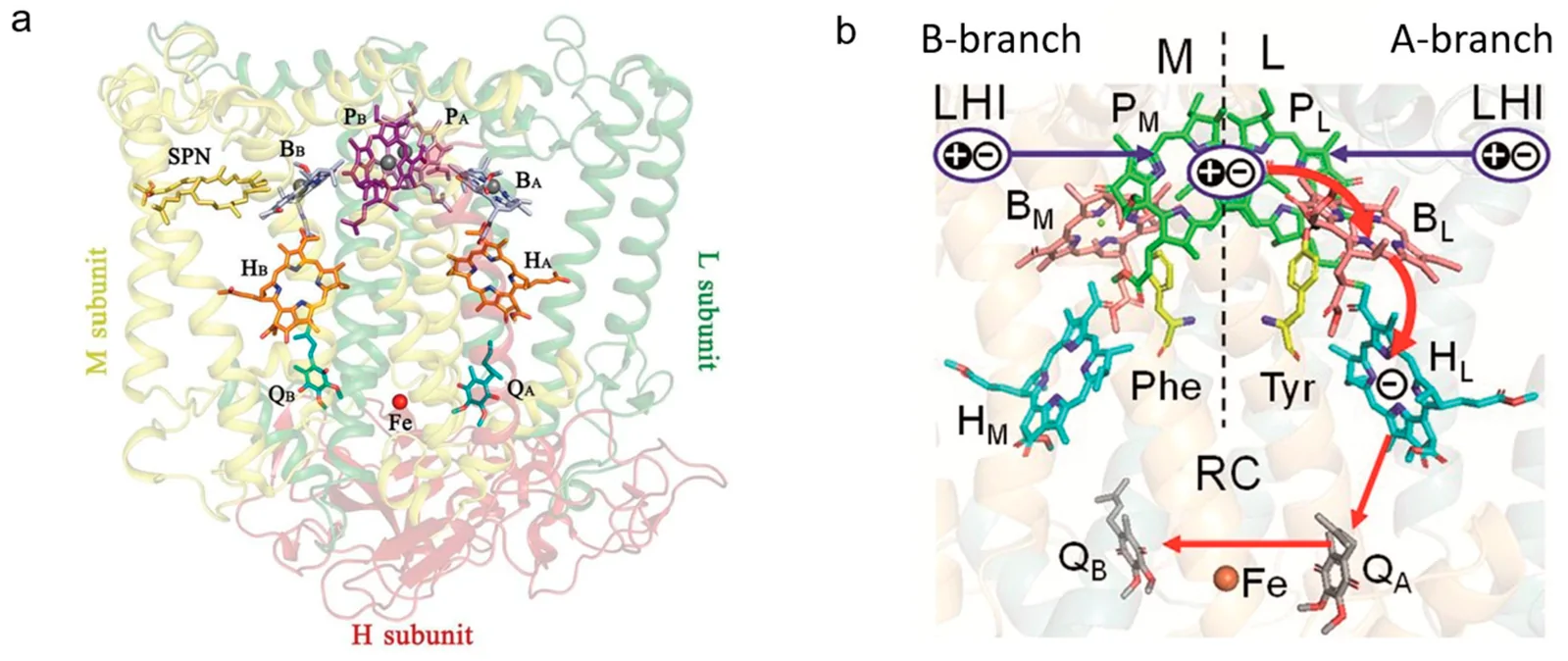
Structure of PbRC from *Rhodobacter sphaeroides* and the unidirectional charge-separation pathway. (**a**) Overall structure of PbRC, an integral membrane protein consisting of ten cofactors encased in a scaffold provided by three polypeptide subunits (backbones shown in light gray) [42]; (**b**) energy-transfer and charge-separation pathways (red arrows) in PbRC [41]. (**a**) reproduced from Ref. [42], under the CC BY 4.0 license. (**b**) adapted and reproduced from Ref. [41], under CC BY 3.0 license.

PbRC is an integral membrane protein complex that provides a highly organized scaffold for light-driven transmembrane electron transfer. As shown in Figure 1 [3,39,41], the high-resolution crystal structures of the reaction centers from *Rhodopseudomonas viridis* and *Rhodobacter sphaeroides* revealed that the complex is composed of three major transmembrane polypeptide subunits, denoted L, M, and H. The L and M subunits each contain five transmembrane α-helices and are arranged around a pseudo two-fold symmetry axis perpendicular to the membrane plane, forming the core scaffold that accommodates the photochemical cofactors. The H subunit contains a single transmembrane helix and a large cytoplasmic domain that caps the L and M subunits, contributing to the stabilization and organization of the cofactor-binding environment. As shown in Figure 1b, the L and M subunits enclose a set of ten cofactors, including four bacteriochlorophylls (BChls), two bacteriopheophytins (BPhe), two ubiquinones, one carotenoid (Car), and a non-heme iron center. The primary electron donor is the special pair of bacteriochlorophylls ($P_{A(L)}$ and $P_{B(M)}$), whose close arrangement enables strong electronic coupling and formation of the excitonically coupled dimer. The remaining cofactors are organized into two quasi-symmetric electron-transfer branches, termed the active A branch and the inactive B branch, consisting of monomeric BChls ($B_{A(L)}/B_{B(M)}$), BPhe ($H_{A(L)}/H_{B(M)}$), and ubiquinones ($Q_{A}/Q_{B}$). Despite this structural pseudo-symmetry, photoinduced electron transfer proceeds predominantly along the A (or L) branch as a result of asymmetrical pigment–protein interactions, leading to formation of the charge-separated state $P^{+}H_{A}^{-}$ and subsequent electron transfer to $Q_{A}$.

The special pair plays a central role in initiating the primary charge-separation process by acting as both an excitonic donor and an electronic gateway to the downstream electron-transfer cofactors [5,7,43]. Structural studies revealed that the two BChl molecules within the special pair are not completely equivalent; differences in pigment orientation, hydrogen bonding, and local electrostatic interactions break the symmetry between the two monomers and modulate the energetics of excitonic and charge-transfer states, regulating excitation transfer, state mixing, and subsequent electron-transfer reactions [43]. More specifically, the local electrostatic environment, dielectric screening, and steric orientations of peripheral pigment substituents, particularly the acetyl and keto groups, act cooperatively to rectify intrapair charge transfer, preferentially stabilizing the $P_{A}^{+}P_{B}^{-}$ configuration. Recent nonadiabatic quantum dynamics simulations demonstrated that specific amino acid-mediated interactions within the protein matrix play a decisive role in establishing the energetic asymmetry required for directional electron transfer [41]. In particular, the Tyr-M210 residue adjacent to the active L branch selectively stabilizes the intermediate charge-transfer state $P^{+}B_{A}^{-}$ through electrostatic interactions, lowering its energy relative to the corresponding inactive-branch intermediate $P^{+}B_{B}^{-}$ (Figure 1b). This amino acid-controlled energetic tuning promotes electron transfer along the L branch while suppressing competing pathways through the M branch. Therefore, the remarkable directionality of charge separation in PbRC arises from the synergistic interplay between the excitonic properties of the special pair, pigment–pigment electronic coupling, and precise protein-mediated modulation of charge-transfer energetics, rather than from structural asymmetry alone.

### 2.2. Excitonic Structure of PbRC

**Figure 2:**
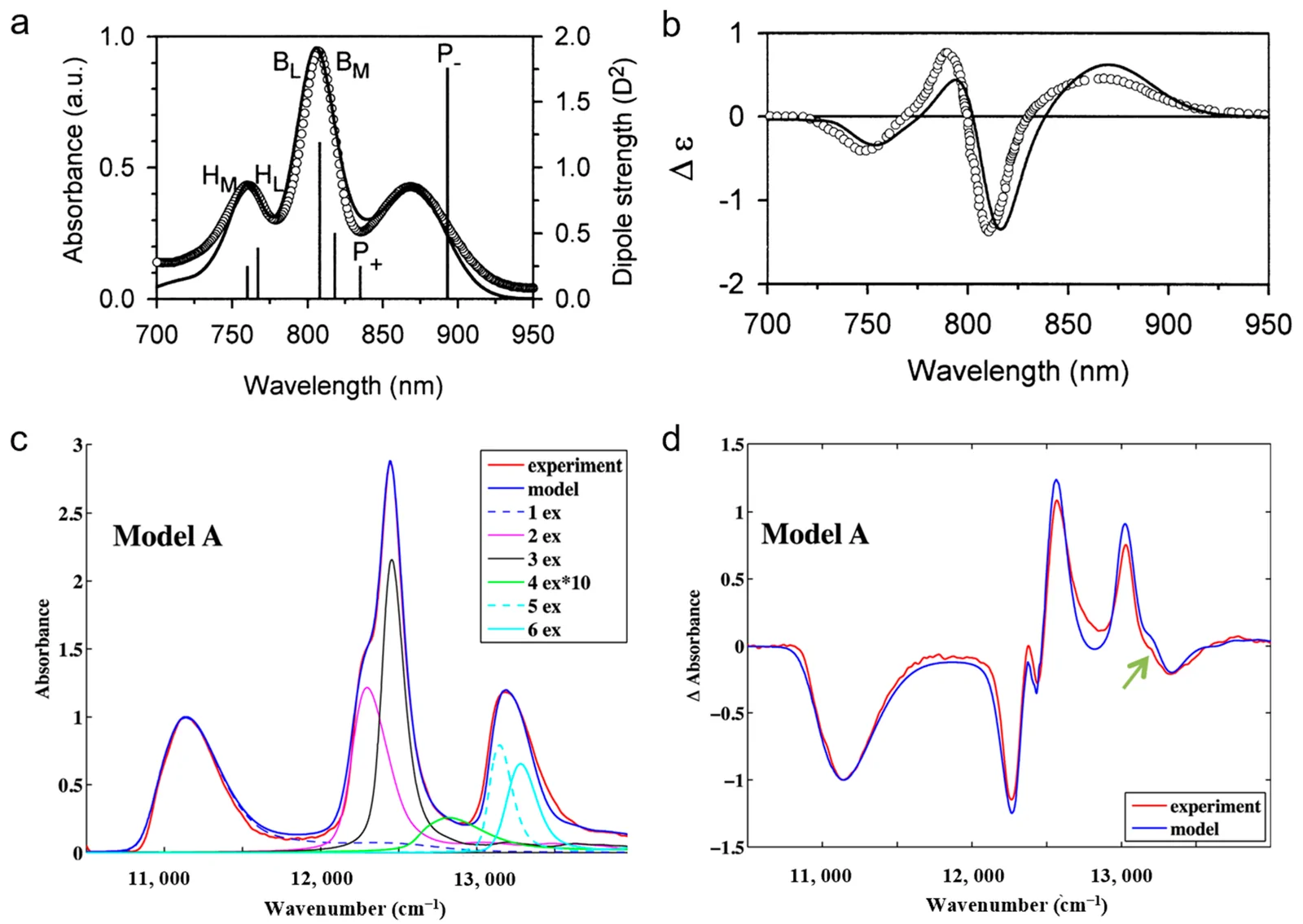
Comparisons of calculated (solid line) and experimental spectra for PbRC reported by the Fleming group [15] (**a**,**b**) and the Abramavicius and Jankowiak groups [45] (**c**,**d**). (**a**) The calculated (solid line) and experimental absorption (circles) spectra for *Rhodobacter sphaeroides* RC at 298 K. The bars indicate the relative intensities and positions of the calculated eigenstates. (**b**) The calculated (solid line) and experimental (circles) circular dichroism spectra for *Rhodobacter sphaeroides* RC at 298 K. (**c**) Absorption spectra of the charge-neutral (CN) WT PbRC with Δ*E_P_* = 500 cm^−1^ and *V_P_* = 750 cm^−1^ [45]. Red and blue lines denote the experimental and calculated spectra, respectively, whereas the other colored lines represent individual calculated excitonic bands. The amplitude of the fourth excitonic band (green line) is magnified tenfold for clarity. (**d**) Transient hole-burning (HB) spectrum (κ_HB_ = κ_CS_ − κ_CN_, red), and the calculated difference absorption spectra (blue) of the charge-separated and CN states of WT PbRC [45]. (**a**,**b**) reprinted with permission from Ref. [15], https://doi.org/10.1021/bi960462i. Copyright © 2001 American Chemical Society. (**c**,**d**) reprinted with permission from Ref. [45], https://doi.org/10.1021/bi9625197. Copyright © 2016 American Chemical Society.

Because of the large oscillator strengths of the $Q_{y}$ transitions, electronic coupling among the closely packed BChls and BPhe produces a series of delocalized excitonic states whose energies are determined by pigment arrangement, electronic interactions, and the surrounding protein environment [10,15,44,45]. For a realistic coupling strength of 625 ${cm}^{-1}$, the special pair forms the dominant excitonic unit, in which coupling between the two BChl molecules generates two exciton states, the lower-energy $P_{-}^{*}$ and higher-energy $P_{+}^{*}$ states. The energy splitting between these states reflects the strength of excitonic coupling within the special pair and provides a sensitive probe of the electronic structure of the reaction center. However, the exact energy and character of the $P_{+}^{*}$ state have remained under debate because of its weak oscillator strength, strong overlap with neighboring excitonic transitions, and possible mixing with charge-transfer configurations. Figure 2 displays the excitonic states calculated by two different groups. These studies placed the $P_{+}^{*}$ state over a broader range of approximately 12,100–12,800 ${cm}^{-1}$ (850–780 nm), depending on the bacterial species, temperature, and experimental conditions [45,46,47]. A more recent theoretical study further suggested that mixing between excitonic and charge-transfer states can substantially modify the energies and oscillator strengths of the special-pair excitons, highlighting the importance of the protein environment in shaping PbRC electronic structure [48]. Using 2DES combined with global analysis, Niedringhaus et al. directly resolved the previously hidden $P_{+}^{*}$ state and assigned it to approximately 11,900 ${cm}^{-1}$ (840 nm) at 77 K, revealing an excitonic state that had been inaccessible by conventional spectroscopy [22]. These results established a detailed excitonic-state framework for PbRCs and demonstrated that the special pair is not a simple two-level system but a coupled exciton–charge-transfer manifold embedded within a protein-controlled electronic environment. The discrepancies among reported $P_{+}^{*}$ energies likely originate from differences in bacterial species, sample environments, and experimental conditions, as well as from variations in theoretical models regarding the extent of exciton–charge-transfer state mixing, which can significantly alter the predicted energies and oscillator strengths of the special-pair excitons.

In contrast, the accessory BChls ($B_{A}$ and $B_{B}$) and BPhe ($H_{A}$ and $H_{B}$) lie at higher excitonic energies of about 12,510 cm^−1^ (799.4 nm), 12,400 cm^−1^ (806.5 nm), 13,180 cm^−1^ (758.7 nm) and 13,420 cm^−1^ (745.2 nm), respectively. These pigments exhibit weaker electronic interactions (i.e., <130 cm^−1^) with one another and with the special pair. Consequently, their excitations are often described as more localized pigment states, although mixing with neighboring cofactors introduces partially delocalized excitonic character. Quantum chemical calculations have shown that the B-associated states possess moderate coupling with the special-pair manifold, enabling efficient excitation transfer toward P, whereas the H states are more weakly coupled and retain a more localized character associated with their role as the primary electron acceptors [15,44]. However, recent theoretical and experiment works have demonstrated that the upper exciton (P) may also mix with $B_{A}$/$B_{B}$ excitations and contributes mainly to excitonic states in the 785–825 nm region [8,10]. For a realistic special-pair coupling of 625 ${cm}^{-1}$, the calculated exciton delocalization lengths of the major states are approximately 2–4 pigments, indicating substantial delocalization across the special pair and accessory BChls [10]. Thus, PbRC electronic structure is best described as a hierarchical excitonic network, consisting of a strongly coupled special-pair core, partially delocalized accessory BChl states, and more localized bacteriopheophytin states.

### 2.3. Primary Charge Separation in PbRC

PbRC has served as a paradigmatic system for understanding how biological photochemical complexes achieve ultrafast and highly efficient charge separation [27,39]. Following photoexcitation, the absorbed energy from antenna is funneled into the excited state within the RC, which initiates electron transfer along the functional A branch. The subsequent electron-transfer cascade generates a long-lived charge-separated state, $P^{+}Q_{A}^{-}$, through sequential electron transfer from the special pair to the accessory $B_{A}$, $H_{A}$, and quinone acceptor ($Q_{A}$). The near-unity quantum yield of this process arises from a delicate balance between electronic coupling, energetic ordering of intermediate states, and protein-mediated stabilization of charge-transfer configurations [49].

**Figure 3:**
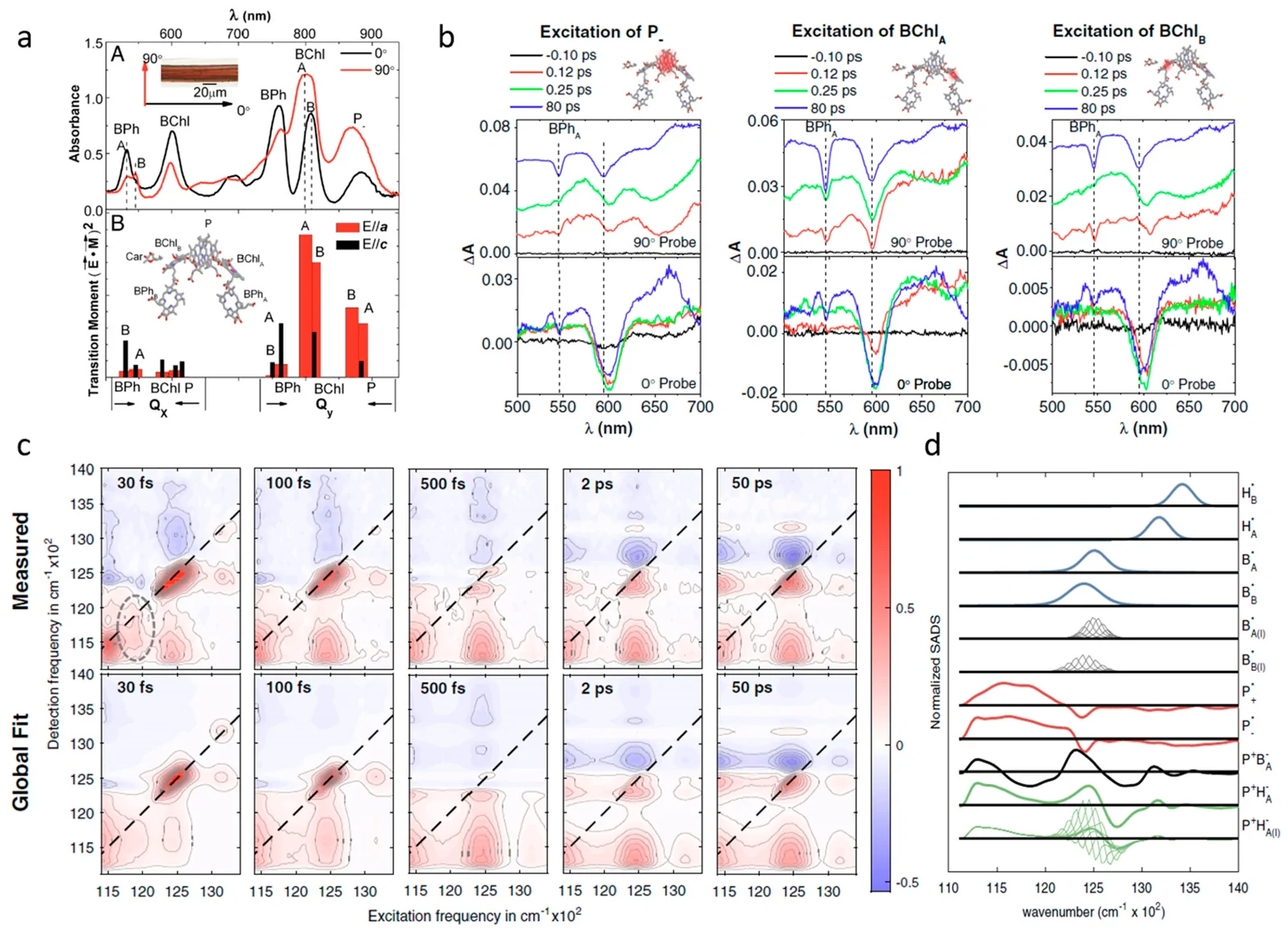
Charge-separation pathways in PbRCs. (**a**) Polarized ground-state optical absorption for a single *Rhodobacter sphaeroides* R26 RC crystal at 100 K [52]. (**Top**) Absorption spectra measured with light polarized perpendicular (90°) and parallel (0°) to the long axis of the crystal. The inset shows a representative photograph of the wild-type (WT) crystals used in the experiments. (**Bottom**) Transition moment projections along the crystal a (red) and c (black) axes, respectively. The inset illustrates the arrangement of the cofactors in RCs, with A and B denoting two branches. (**b**) Pump-wavelength-dependent patterns of transient absorption (TA) spectra in a single *Rhodobacter sphaeroides*-wt crystal at 100 K [52]. The pump was adjusted to 900 nm (90°), 790 nm (90°), and 824 nm (0°). (**Top**) TA spectra probed at 90° with respect to the crystal c axis and the spectra are offset for clarity. The vertical lines are placed at 545 and 595 nm. (**Bottom**) TA spectra probed at 0°. (**c**) Absorptive 77-K 2DES spectra (**Top**) from the W(M250)V mutant BRC in 50/50 buffer/glycerol with magic-angle polarization at different waiting times [22]. Two-dimensional spectra (**Bottom**) reconstructed from excitation-dependent global fits to the single-pathway model. The dashed oval in the 30 fs measured spectrum indicates the location of the $P_{+}^{*}$ state, which shows a weak diagonal signal and cross-peak from internal conversion to $P_{-}^{*}$. (**d**) Optimized species-associated difference spectra obtained from the global fit of 2DES [22]. $H_{B}^{*}$, $H_{A}^{*}$, $B_{A}^{*}$, and $B_{B}^{*}$ have the same form as the linear absorption fit. $B_{A(I)}^{*}$ and $B_{B(I)}^{*}$ show a subset of the inhomogeneous Lorentzian lineshapes. The $P_{+}^{*}$ and $P_{-}^{*}$ spectra were constrained based on fits to the data at an excitation frequency of 11,900 cm^−1^. The $P^{+}H_{A}^{-}$ and $P^{+}H_{A(I)}^{-}$ states were derived from the $B_{A(I)}^{*}$ and $B_{B(I)}^{*}$ Lorentzian bands, and the $P^{+}B_{A}^{-}$ difference spectrum is calculated by linear least squares. (**a**,**b**) are adapted from Ref. [52], with permission from PNAS. (**c**,**d**) are adapted from Ref. [22], with permission from PNAS.

Early ultrafast spectroscopic studies established that primary charge separation in PbRCs proceeds through intermediate charge-transfer states, although the identity of the initial photochemical pathway remained debated. The conventional model proposed that excitation of the special pair generates $P^{*}$, followed by sequential electron transfer through the accessory B and H cofactors, forming $P^{+}B_{A}^{-}$ and subsequently $P^{+}H_{A}^{-}$ states [50,51]. However, the strong spectral overlap among excited-state absorption, stimulated emission, and charge-transfer signatures prevented unambiguous identification of the earliest electron-transfer steps, leading to alternative models involving direct $P^{*}$→${BPh}_{A}$ electron transfer through superexchange mechanisms or contributions from higher-energy accessory pigment excitations [13,49]. In particular, van Brederode et al. suggested [13] the existence of multiple ultrafast photochemical routes, including pathways that do not directly involve the special pair as the initially excited state. Huang et al. provided direct evidence for such cofactor-specific pathways using polarization-selective ultrafast spectroscopy of single *Rhodobacter sphaeroides* reaction-center crystals [52] (Figure 3a,b). They demonstrated that selective excitation of $B_{B}$ rapidly relaxes to a localized $P^{*}$ state before charge separation, whereas excitation of $B_{A}$ can initiate an alternative charge-separation pathway through a $B_{A}^{+}H_{A}^{-}$ state that does not evolve from $P^{*}$. The remaining $B_{A}$-excited population can also relax to $P^{*}$, followed by the conventional sequential electron-transfer pathway. These results suggested that the bacterial reaction center cannot be described by a single $P^{*}$-initiated electron-transfer pathway, but rather as a multicofactor excitonic system capable of supporting excitation-dependent charge-separation routes.

A recent 2DES study has provided more direct evidence supporting a sequential charge-separation model [22] (Figure 3c,d). Using broadband 2DES combined with multi-excitation global analysis, Niedringhaus et al. [22] resolved the excitonic states of PbRC and extracted the spectral signatures of the charge-separated intermediates. Their analysis demonstrated that the observed dynamics can be described by a two-step pathway in which excitation of P leads first to the formation of $P^{+}B_{A}^{-}$, followed by conversion to $P^{+}H_{A}^{-}$. Importantly, the analysis did not require an additional charge-separation pathway originating from $B_{A}^{*}$, suggesting that the special pair remains the primary electron donor despite the strong electronic coupling among pigments. This work helped clarify previous conflicting interpretations from transient absorption experiments by demonstrating that excitation-frequency-resolved measurements are essential for distinguishing overlapping kinetic pathways.

The timescales of the individual electron-transfer steps also depend on temperature, species, and structural perturbations [9,13,22,52,53]. In *Rhodobacter sphaeroides* reaction centers, the formation of the primary charge-separated state $P^{+}H_{A}^{-}$ generally occurs within several picoseconds. At cryogenic temperatures, the P* →$P^{+}B_{A}^{-}$ transition occurs on approximately 1–2 ps, followed by electron transfer from $B_{A}^{-}$ to $H_{A}$ within approximately 0.5–1 ps. The subsequent transfer from $H_{A}^{-}$ to $Q_{A}$ is substantially slower, occurring on hundreds of picoseconds, reflecting the stabilization and reorganization required for long-distance electron transfer.

### 2.4. Coherence in PbRC

The ultrafast primary charge-separation process in PbRCs occurs on a timescale comparable to electronic dephasing and nuclear vibrational motions, motivating extensive investigations into whether and how coherent dynamics influence excitation transfer and electron-transfer processes. Early femtosecond transient absorption measurements by Vos et al. revealed pronounced low-frequency vibrational coherence at approximately 15 and 77 cm^−1^ in *Rhodobacter sphaeroides* RC, which were interpreted as coherent nuclear wave-packet motions coupled to the excited-state dynamics of the reaction center [12,54]. Subsequent polarization-controlled two-color coherence photon echo measurements have demonstrated that vibrational coherence at 685 cm^−1^ associated with the B ground electronic state may modulate energy-transfer dynamics from H to B [30]. Another 2DES study of mutants revealed [55] that perturbation of the special-pair environment can modify the vibronic coherence but not the energy-transfer dynamics. Thus, whether the coherence plays a role in energy transfer and charge separation remains unresolved [37,38]. This question is particularly important for PbRCs because the special pair, accessory B, and H form a strongly coupled excitonic system in which electronic and vibrational degrees of freedom are intrinsically mixed.

**Figure 4:**
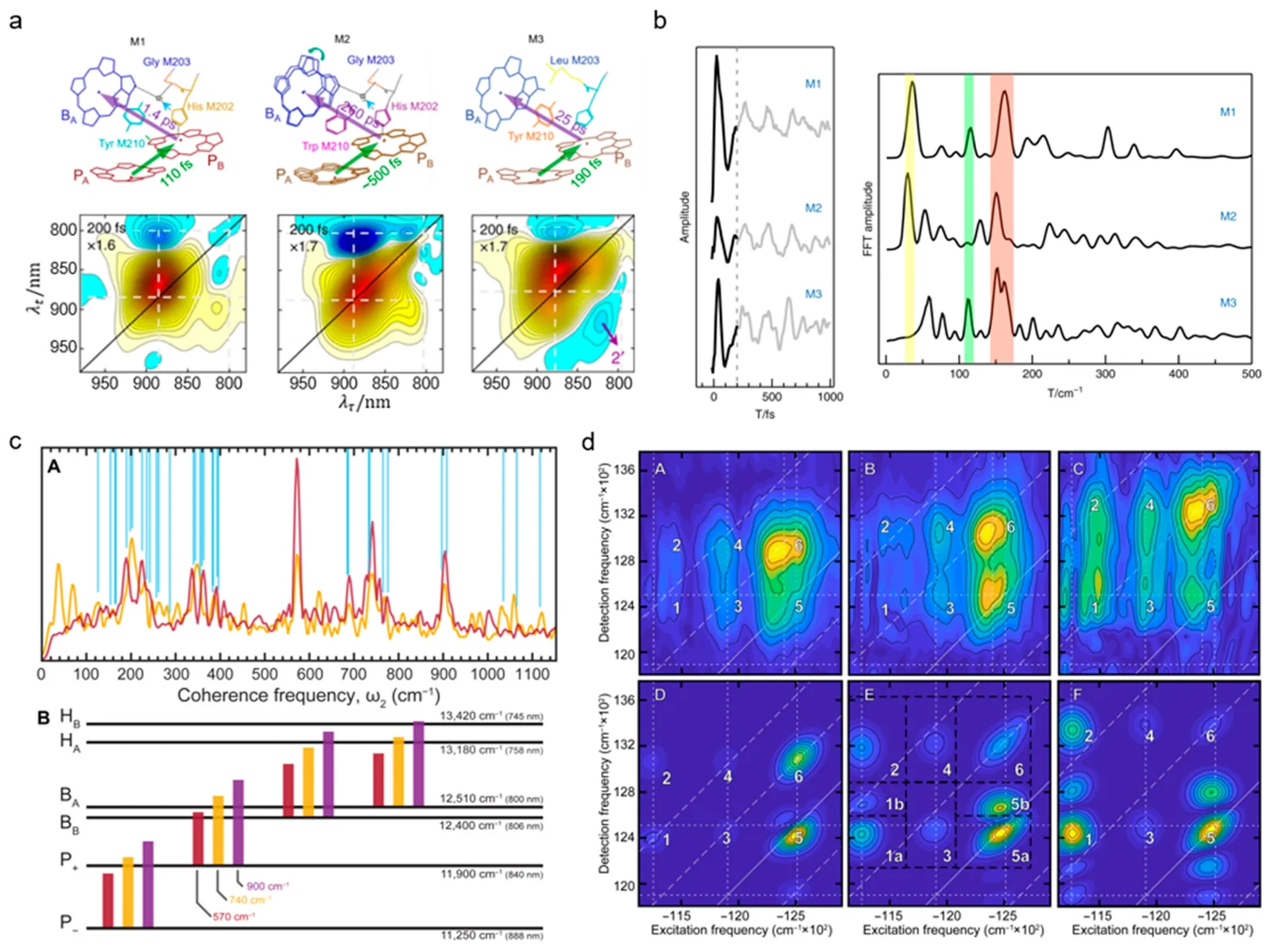
Coherent dynamics associated with primary charge separation in different PbRC mutants. (**a**) Scheme for primary charge separation in mutant RCs M1 (**left**), M2 (**middle**) and M3 (**right**) [9]. M1 is a *Rhodobacter sphaeroides* RC mutant in which Ala at position M260 is replaced by tryptophan (AM260W). In M2, B_A_ and P are slightly tilted relative to one another and the weak hydrogen bond (gray dotted line) between Tyr-M210 and P_B_ is disrupted by the replacement of tyrosine at M210 position with a larger tryptophan. In M3, the water molecule linking P_B_ and B_A_ via a hydrogen-bond interaction (black dotted lines) is removed upon the replacement of the glycine at the M203 position with leucine. The green and purple arrows show the two steps of primary charge separation with time constants obtained from a previous study. Also shown are absorptive 2D spectra at the waiting time of 100 fs. (**b**) Quantum beats and Fourier transform (FT) power spectra [9]. The upper, middle, and bottom traces on the left represent real-part time traces at the cross-peak of (P*, $P_{A}^{+}P_{B}^{-}$) for M1, M2, and M3, respectively, after subtraction of multiexponential population dynamics. The right panel shows the summary FT power spectra of the residual oscillations over 0–2 ps. (**c**) Frobenius spectra for the W(M250)V PbRC (red) and BChl a (gold) [11]. Blue lines indicate vibrational modes of BChl a and BPhe that lie within the experimental resolution (9.8 cm^−1^) of the most prominent peaks in the Frobenius spectra. The indicated excitonic energy levels demonstrate how several prominent coherence modes are in quasi-resonance with the energetic gap between excitonic levels. (**d**) Experimental (**top**) and simulated (**bottom**) complex rephasing coherence maps [11] for ω_2_ = +570, +740, and +900 cm^−1^ featuring the prominent signatures (labeled 1 to 6) of vibronic coherence between special pair and accessory BChl sites. (**a**,**b**) adapted from Ref. [9] under the CC BY 4.0 License. Panels (**c**,**d**) adapted with permission from Ref. [11]. Modified from V. R. Policht et al., *Science Advances*, doi:10.1126/sciadv.abk0953, AAAS.

More direct insight into coherence during charge separation was obtained by measuring 2DES spectra of mutants. Ma et al. [9,25] investigated *Rhodobacter sphaeroides* RCs and demonstrated that both electronic and vibrational coherences are involved during primary charge separation (Figure 4a,b). They identified short-lived electronic coherence at 160–260 cm^−1^ between P* and $P_{A}^{+}P_{B}^{-}$, together with vibrational coherences associated with pigment and protein motions during the formation of the primary charge-separated state. The authors attributed the mutation-dependent changes in the amplitudes of electronic coherence to perturbations of cofactors’ configurations and/or the absence of $P_{A}^{+}P_{B}^{-}$. In contrast, Policht et al. [11] performed 2DES on the W(M250)V mutant and found that the Frobenius spectrum of PbRC is almost identical to that of the BChl a (Figure 4c), suggesting that vibrational coherence may dominate in the observed coherent dynamics. More importantly, by combining broadband 2DES and theoretical simulations (Figure 4d), these authors revealed coherence transfer of three vibrational modes at 570 cm^−1^, 740 cm^−1^ and 900 cm^−1^ between vibronic states comprising B excitons and the special pair. Furthermore, the coherence map provided insights into the hidden vibronic and excitonic structures of the special pair in PbRC. These findings provide a molecular basis for the coherent oscillations observed in PbRC.

Together, these studies establish that coherent electronic and vibrational dynamics are intrinsic features of the PbRC excitonic landscape. However, whether coherence directly enhances the efficiency of charge separation remains under debate. The high efficiency and directionality of charge separation in PbRC are generally attributed to the optimized energetic landscape, pigment–pigment coupling, and protein-mediated tuning of charge-transfer states, while coherence may contribute by modulating interactions among excitonic, vibrational, and charge-transfer states during the earliest stages of photochemistry.

## 3. Charge Separation in HbRC

### 3.1. Structure of HbRC

**Figure 5:**
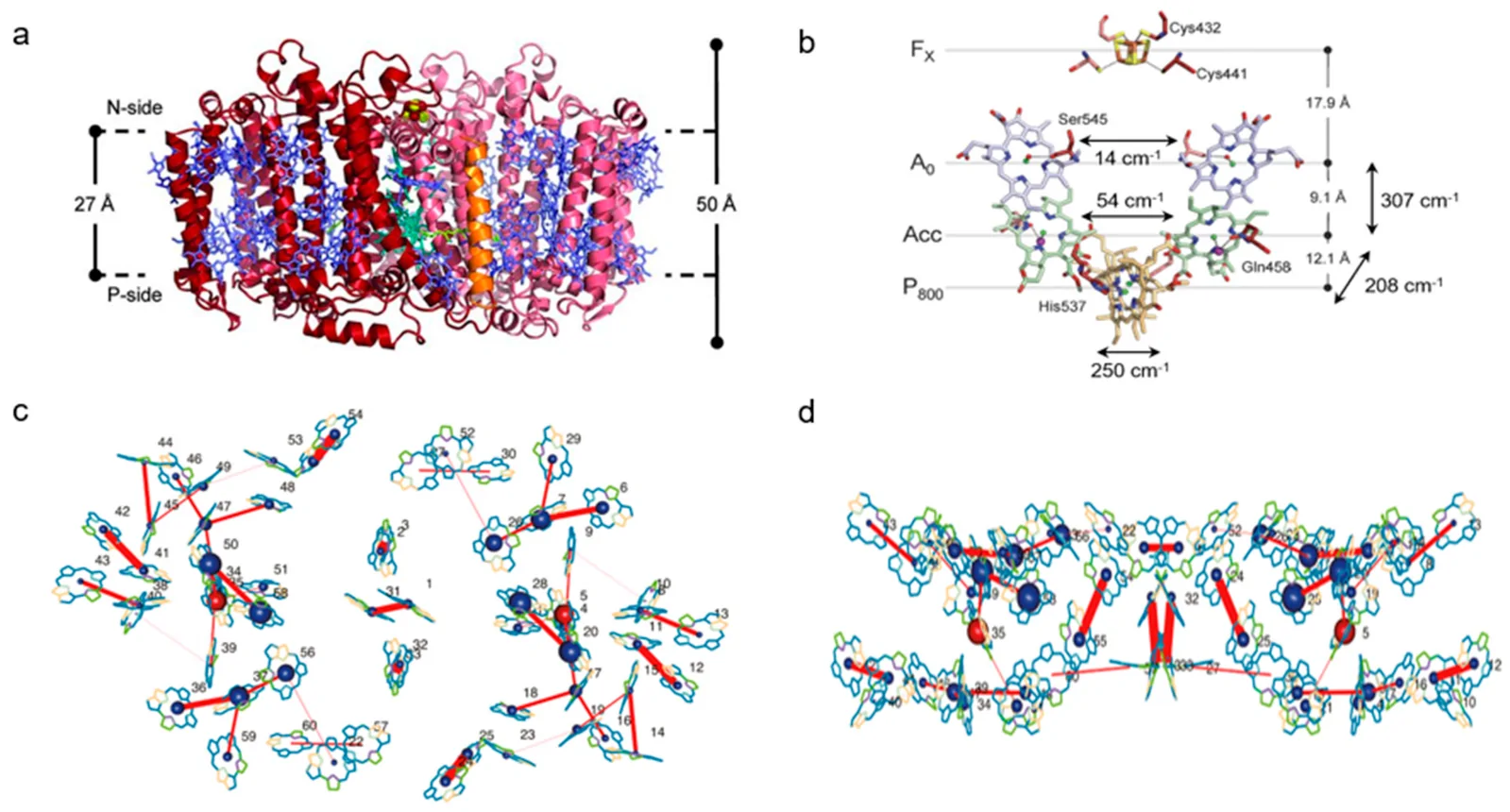
Structure, cofactor organization, and excitonic properties of HbRC. (**a**) The overall structure of HbRC viewed from the N-side within the membrane [28]. The two PshA polypeptides are colored in red and pink. PshX subunits in orange. Cofactors are shown as stick models and colored teal (ET), blue (antenna), and lime (carotenoids). The [4Fe-4S] cluster is shown as red (Fe) and yellow (S) spheres. The BChl and Chl tails have been truncated for clarity. (**b**) Cofactor arrangement of the ET chain [28]. Cofactor carbons are colored differently to ease viewing: P800 (orange), Acc (green), and A_0_ (blue). (**c**,**d**) Top (**c**) and side (**d**) views of the arrangement of BChl g, BCh g′, and Chl a_F_ and the calculated excited-state distribution of the lowest excitonic state of H. modesticaldum HbRC [33]. Sizes of spheres at the center of the pigments indicate the distribution probabilities of the excitation residing on individual pigments. Red lines indicate pathways with the highest calculated excitation-energy-transfer probabilities between pigments. (**a**,**b**) adapted with permission from Ref. [28]. Modified from C. Gisriel et al., *Science*, https://doi.org/10.1126/science.aan5611, AAAS. Figure 5c,d reproduced with permission from Ref. [33], https://doi.org/10.1021/acs.jpcb.8b08014. Copyright © 2018 American Chemical Society.

Heliobacteria were discovered serendipitously and first identified as a distinct group of anoxygenic phototrophs in the early 1980s by Gest and Favinger [56]. The first HbRC purification was achieved by Trost and Blankenship using detergent-solubilized membranes and sucrose-density-gradient ultracentrifugation [57], and was later improved by replacing the gradient step with sequential ion-exchange chromatography [58]. Subsequent biochemical and structural studies [4,28] revealed that they contain a unique Type I photosynthetic reaction center that uses BChl g as the primary pigment and transfers electrons to a [4Fe–4S] iron–sulfur cluster. Unlike the heterodimeric RCs of purple bacteria, photosystem II (PSII), and photosystem I (PSI), HbRC was proposed to preserve an ancestral homodimeric architecture, providing an important evolutionary link between primitive and modern photosynthetic reaction centers. This hypothesis was confirmed by the first high-resolution crystal structure of HbRC from *Heliobacterium modesticaldum*, determined by Gisriel et al. [28] at a 2.2 Å resolution using X-ray crystallography. The structure revealed a C_2_-symmetric homodimer consisting of two PshA core proteins and two small PshX transmembrane subunits, representing the first atomic-resolution structure of a homodimeric photosynthetic RC (Figure 5a).

The HbRC core contains two functional regions: an N-terminal antenna domain that binds the majority of the light-harvesting BChls and a C-terminal electron-transfer (ET) domain that accommodates the redox cofactors responsible for primary charge separation [28]. As shown in Figure 5b, the ET domain is formed by the central transmembrane regions of the PshA homodimer and contains the primary electron donor P800, intermediate electron-transfer cofactors, and the terminal [4Fe–4S] acceptor cluster F_X._ P800 consists of a pair of BChl g′ molecules located near the twofold symmetry axis, while electron transfer proceeds through additional cofactors, including accessory BChl g and 8^1^-hydroxychlorophyll a (8^1^-OH-Chl a) molecules, toward the F_X_ cluster coordinated by conserved cysteine residues from both PshA subunits. The spatial arrangement and inter-cofactor distances among the special pair, accessory pigments, and electron acceptors in HbRC are broadly similar to those in PSI. However, the spectrally distinct absorption of the primary electron acceptor, 8^1^-OH-Chl a, in HbRC makes it a particularly tractable model for elucidating charge-separation mechanisms relevant to PSI, where the absorption bands of the antenna and core reaction-center pigments are strongly congested. In contrast to the PSI RC, HbRC lacks a bound quinone cofactor between the primary donor and terminal acceptor, indicating a shorter electron-transfer pathway from the Chl cofactors to F_X_.

In contrast to PbRC and PSII RC, HbRC features a substantially longer distance between the special pair and the primary electron acceptor, A_0_ [4]. This arrangement shortens the distance between Fx and A_0_ and facilitates electron transfer between these two cofactors. Additionally, the accessory BChl in HbRC adopts a perpendicular orientation relative to the membrane plane (and thus the static electric field), whereas it aligns parallel in PbRC and the PSII RC. This perpendicular arrangement may facilitate electron transfer from the special pair to A_0_ while stabilizing the intermediate charge-separated state. Moreover, the symmetric arrangement of the ET cofactors and the absence of branch specialization suggest that HbRC retains features associated with ancestral homodimeric RCs [4]. Readers interested in the evolutionary history of RCs are referred to Ref. [4].

### 3.2. Excitonic Structure of HbRC

The nearly symmetric arrangement of P800, Acc, and A_0_ in the ET domain of HbRC gives rise to an excitonic landscape distinct from that of the pseudo-symmetric PbRC. Structure-based theoretical calculations [33,34] have shown that the closely spaced pigments of the HbRC core form strongly coupled excitonic states rather than isolated pigment transitions. The close packing of P800, Acc, and A_0_ pigments generates substantial electronic couplings (Figure 5b), producing excitonic states delocalized over multiple cofactors. Within the ET domain, low-energy excitonic states contain significant contributions from the P800 special pair, whereas higher-energy states involve increasing contributions from Acc and A_0_ pigments, establishing an excitonic energy hierarchy that connects excitation redistribution with the formation of charge-transfer states. Comparisons with PSI further demonstrated that HbRC possesses a distinct excitonic organization arising from its smaller pigment network and unique BChl *g*-based architecture.

More recent first-principles calculations have revealed that the HbRC excitonic manifold is strongly coupled to charge-transfer states and is modulated by the protein environment [31,59,60,61]. Using quantum chemical calculations combined with molecular dynamics simulations, Brütting et al. [31,59] showed that the bright excitations mainly originate from coupled BChl g $Q_{y}$ transitions, while several low-lying charge-transfer states involving Acc and A_0_ pigments are embedded within the excitonic manifold. Importantly, charged amino acids near A_0_ selectively stabilize A_0_-centered charge-transfer states, shifting the ${Acc}^{+}{A_{0}}^{-}$ state below the bright excitons and providing a microscopic explanation for efficient primary charge separation. A subsequent first-principles study by Ertürk et al. [61] examined the electron-transfer domain using a screened range-separated hybrid functional approach combined with a polarizable continuum model. Their calculations revealed that the lowest-energy optical excitations arise from coupled BChl $g/g^{\prime }$ transitions, while the relative energies and electronic characters of the excited states are strongly influenced by the electrostatic environment of the protein matrix.

### 3.3. Energy Transfer and Charge Separation in HbRC

**Figure 6:**
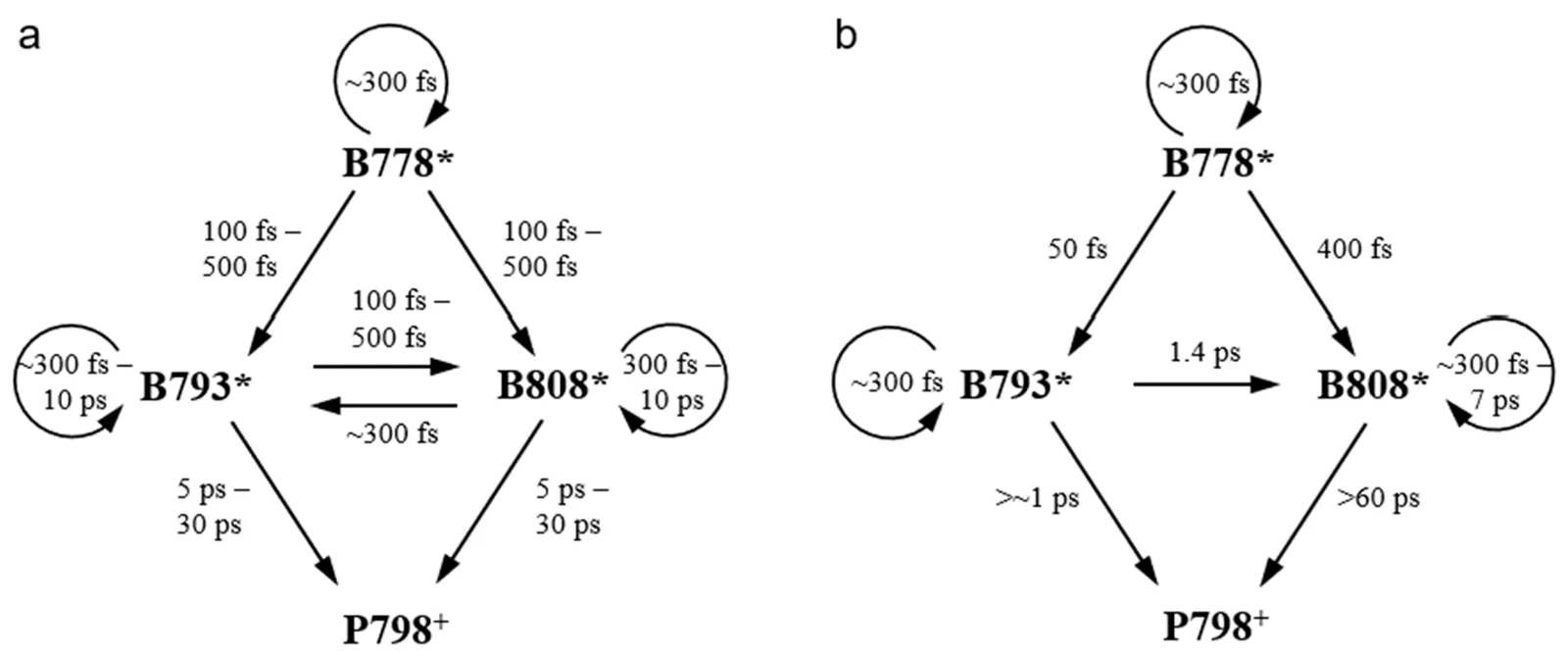
Proposed energy-transfer pathways between the different BChl g pools and trapping by P798 [64,65] (**a**) at room temperature and (**b**) at 20 K. B778*, B793* and B808* denote excited states comprised of BChl g molecules peaked at 778 nm, 793 nm and 808 nm, respectively. The circular arrows refer to energy transfer between individual pigments of the same pool. (**a**) reprinted with permission from Ref. [65], https://doi.org/10.1021/bi960462i. Copyright © 1996 American Chemical Society. (**b**) reprinted with permission from Ref. [64], https://doi.org/10.1021/bi9625197. Copyright © 1997 American Chemical Society.

Excitation-energy transfer and charge separation in HbRC occur within a highly coupled pigment network, where rapid energy redistribution competes with photochemical trapping. Early ultrafast studies [62,63] on *Heliobacillus mobilis* showed that excitation equilibration among the BChl g antenna pigments occurs within subpicosecond-to-picosecond timescales, followed by decay of antenna excitation and formation of the charge-separated state with an apparent lifetime of approximately 25–27 ps. More detailed kinetic analyses [64,65] revealed multiple components associated with excitation trapping, including approximately 5 and 30 ps processes, suggesting that charge separation can initiate before complete equilibration of the antenna manifold [64,65] (Figure 6). Under a trap-limited model, in which the primary donor (denoted P798 in early studies) is assumed to be thermally equilibrated with the antenna, the intrinsic charge-separation time was estimated to be approximately 1.2 ps, although this value depends on the kinetic model and differs from the experimentally observed trapping lifetime. At cryogenic temperatures, selective excitation experiments further revealed heterogeneous energy-transfer pathways among spectrally distinct BChl g pools, with transfer from BChl g 778 to BChl g 793 occurring within approximately 50 fs and subsequent redistribution toward lower-energy BChl g 808 states occurring on subpicosecond-to-picosecond timescales [65]. Importantly, excitation trapping could occur directly from higher-energy antenna states, indicating that excitation redistribution and photochemical trapping are competing processes rather than a simple sequential downhill energy funnel. More recently, Orf et al. demonstrated experimentally that the lowest-energy antenna sites of the complete HbRC are associated with PshX-bound BChl g pigments rather than P800. Low-temperature difference spectroscopy assigned absorption losses at 813 and 795 nm to these pigments, with the 813 nm feature tentatively attributed to an excitonically coupled dimer, i.e., BChl g-1006 bound by PshA and BChl g-102 bound by PshX. Excitation from this low-energy antenna must therefore undergo entropy-assisted uphill transfer to the P800 reaction-center trap [66].

The primary charge-separation mechanism of HbRC has been progressively clarified through spectroscopy [18,24], mutagenesis [35], and theoretical calculations [31,59,61]. Early studies assigned the approximately 25 ps component to excitation trapping and formation of $P_{800}^{+}A_{0}^{-}$, followed by electron transfer from $A_{0}^{-}$ to the $F_{X}$ cluster with a lifetime of approximately 600–700 ps. When forward electron transfer was blocked, the $P_{800}^{+}A_{0}^{-}$ state recombined in approximately 20 ns, while thermodynamic measurements showed that electron transfer from A_0_ to $F_{X}$ is energetically favorable [18,36,67]. Mutagenesis of the A_0_ binding environment further demonstrated that A_0_ is essential for efficient charge separation [35]. Replacement of the Ser545 axial ligand with methionine abolished A_0_ binding and eliminated native charge separation, whereas mutations that altered the A_0_ site energy accelerated both forward electron transfer and recombination but reduced the overall quantum yield, indicating that the native protein environment optimizes the balance between transfer rates and photochemical efficiency.

**Figure 7:**
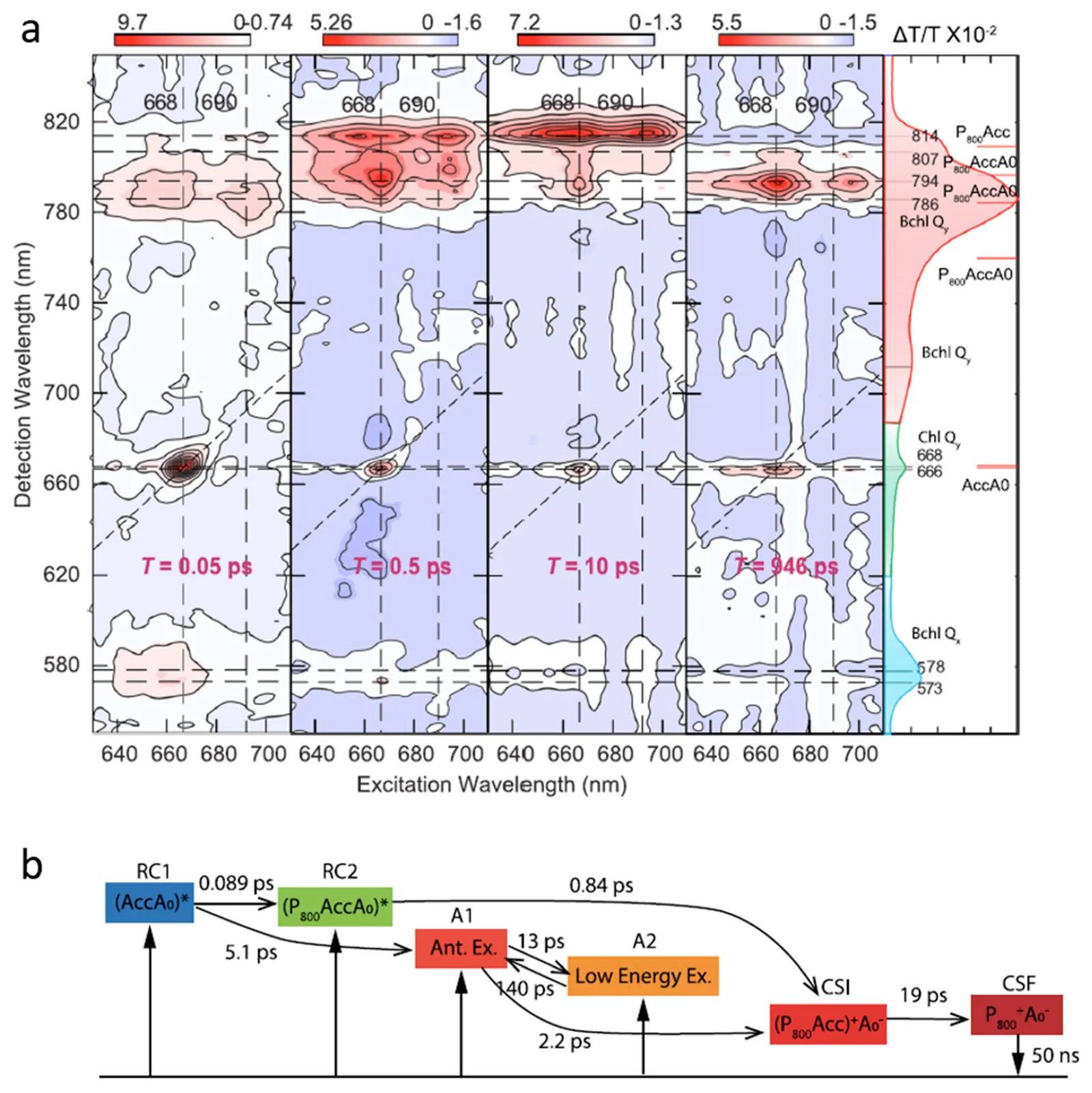
(**a**) 2DES absorptive spectra at 0.05, 0.5, 10, and 946 ps [24]. Alongside the 2D absorptive spectra are the experimental absorption spectrum and the simulated stick spectrum (red lines) reported by Kimura et al. [33]. Dashed lines in the 2DES and the gray lines in the absorption spectrum indicate the various peak positions discussed in the main text. The experimental absorption spectrum is divided into three color-coded regions based on their dominant contributions (i.e., red: the BChl Q_y_ band; green: the Chl Q_y_ band; blue: the BChl Q_x_ band). The contour levels are −1:0.1:1 of the maximum amplitude. (**b**) The kinetic model in which CS proceeds via a two-step mechanism with A_0_ as the primary electron acceptor, independent of whether the initial excitation occurs within the RC or antenna domains [24]. The vertical arrows indicate species that can be generated by direct photoexcitation. Images reproduced from Ref. [24], under the CC BY 4.0 License.

Multispectral two-dimensional electronic spectroscopy provided a detailed experimental description of the primary charge-separation pathway [24]. As shown in Figure 7, global target analysis identified relaxation of the reaction-center exciton manifold within approximately 0.09 ps, formation of a charge-separated intermediate (CSI) within approximately 0.84 ps, and evolution of CSI into the final $P_{800}^{+}A_{0}^{-}$ state within approximately 19 ps. The electron was assigned to $A_{0}$, whereas the distribution of the positive charge remains under debate. The spectroscopic analysis suggested a hole distributed over the coupled $P_{800}Acc$ region [24], while first-principles calculations including $A_{0}$ and the surrounding protein environment favored an ${Acc}^{+}A_{0}^{-}$ as the intermediate [31]. Earlier calculations restricted to the P800–Acc subsystem predicted higher-energy ${P800}^{+}{Acc}^{-}$ charge-transfer states and therefore could not determine the role of $A_{0}$, highlighting the importance of including the complete ET domain and protein environment [59]. Two subsequent studies employed screened range-separated hybrid calculations to estimate the nonadiabatic charge-separation rates within Fermi’s golden rule (FGR) framework based on the subsystems without [60] and with [61] the inclusion of $A_{0}$. These studies provide a useful theoretical basis for comparing experimentally observed and theoretically predicted charge-separation pathways [60,61]. Overall, current evidence supports a model in which excitation energy is redistributed through a coupled antenna–reaction-center excitonic network, followed by $A_{0}$-initiated charge separation and subsequent electron transfer to $F_{X}$, while the exact electronic character and charge distribution of the earliest radical pair remain key unresolved questions.

## 4. Possible Evolutionary Origins of Structural Differences and Their Relevance to Charge Separation

A previous evolutionary analysis [4] proposed that the ancestral photosynthetic RC was homodimeric, with two equivalent electron-transfer branches and possibly loosely bound quinones. In this model, the early divergence of Type I and Type II RCs involved different strategies for optimizing electron transfer to the terminal acceptor: Type I RCs acquired the [4Fe–4S] cluster $F_{X}$, whereas Type II RCs underwent heterodimerization and functional differentiation of the two cofactor branches. The emergence of $F_{X}$ was accompanied by substantial remodeling of the Type I electron-transfer chain, including replacement of (B) Pheo by Chl a-type primary acceptors and reorientation of the accessory (B)Chl. These changes position $A_{0}$ farther from the special pair but closer to $F_{X}$, while the strongly reducing $A_{0}^{-}$ state provides the driving force required for electron transfer to the low-potential Fe–S cluster. The incorporation of epimeric forms of the accessory pigments into the special pair is thought to relieve steric constraints. Thus, evolutionary changes in cofactor identity, orientation, and terminal acceptors may have contributed directly to the distinct charge-separation pathways of Type I and Type II RCs. The subsequent diversification of antenna organization and photoprotective mechanisms likely further adapted these RCs to their different physiological environments.

The resulting structural differences between Type I and Type II RCs are directly relevant to their primary charge-separation mechanisms. In the heterodimeric PbRC, the special pair P, accessory $B_{A}$, and $H_{A}$ form a relatively compact electron-transfer chain. The accessory BChl is oriented largely parallel to the membrane plane, and the center-to-center separation between P and $H_{L}$ is approximately 16.8 Å [3,4]. These geometrical features facilitate strong electronic coupling between neighboring cofactors and are consistent with the rapid sequential pathway $P^{*}\to P^{+}B_{A}^{-}\to P^{+}H_{A}^{-}$, in which the first charge-separated intermediate is formed with a time constant of 2.2 ps in wild-type *Rhodobacter capsulatus*, followed by rapid electron transfer to $H_{A}$ with a time constant of 0.8 ps [22]. Geometry alone, however, cannot explain the almost exclusive use of the L branch because the two branches remain approximately pseudo-symmetric. Directionality instead arises primarily from asymmetric protein electrostatics; in particular, Tyr-M210 stabilizes $P^{+}B_{A}^{-}$, thereby lowering the energy of this intermediate and promoting L-branch charge separation [41].

HbRC preserves an almost perfectly $C_{2}$-symmetric electron-transfer architecture but adopts a markedly different cofactor geometry [28]. The accessory BChl g pigments are oriented more nearly perpendicular to the membrane plane, which moves $A_{0}$ farther from P800 and closer to $F_{X}$; the P800–$A_{0}$ center-to-center separation is approximately 19.3 Å, nearly 2.5 Å longer than the corresponding P–H separation in PbRC. Such an arrangement would be expected to weaken direct electronic coupling between P800 and $A_{0}$ and is consistent with a slower and symmetric charge-separation pathway. Multidimensional spectroscopy indicates that a reaction-center exciton first forms an A_0_-centered charge-separated intermediate within approximately 0.84 ps, followed by conversion to $P_{800}^{+}A_{0}^{-}$ in approximately 19 ps [24]. The sub-picosecond initial step is consistent with local charge separation between the closely positioned electron donor and acceptor, whereas the subsequent slower component can be associated with redistribution of the positive charge toward P800 across the larger P800–A_0_ separation. Thus, the perpendicular orientation of Acc and increased donor–acceptor span in Type I RCs may have evolved together with the introduction of the low-potential Fe–S acceptor chain, producing a charge-separation mechanism distinct from the compact, energetically asymmetric pathway of PbRC. More generally, the comparison suggests that evolutionary changes in cofactor identity, orientation, separation, and protein electrostatics did not merely modify RC structure but reshaped the electronic coupling and energetic landscapes that govern primary charge separation.

## 5. Summary and Outlook

Photosynthetic reaction centers provide highly optimized natural systems for understanding ultrafast and efficient solar-energy conversion. Photosynthetic reaction centers provide highly optimized natural systems for understanding ultrafast and efficient solar-energy conversion. Comparison of PbRC and HbRC reveals two distinct strategies for controlling primary charge separation within a broadly conserved two-branch architecture. In PbRC, strong excitonic coupling within the special pair is combined with protein-induced energetic asymmetry that favors electron transfer along a single active branch. In contrast, the homodimeric HbRC retains two structurally equivalent electron-transfer branches and employs a distinct arrangement of BChl and Chl cofactors that supports charge separation likely involving an A_0_-centered intermediate. The different cofactor geometries, electronic couplings, and energetic landscapes of these two systems therefore illustrate how related RC architectures can support markedly different charge-separation mechanisms.

Viewed from an evolutionary perspective, these differences suggest that changes in cofactor identity, orientation, and separation were closely coupled to changes in terminal electron acceptors and protein electrostatics. In Type II RCs, heterodimerization and asymmetric stabilization of charge-transfer intermediates enabled highly directional electron transfer through a compact cofactor chain. In Type I RCs, the incorporation of low-potential Fe–S acceptors was accompanied by reorganization of the electron-transfer cofactors, including reorientation of the accessory (B)Chl and increased separation between the special pair and A_0_. These structural modifications reshape electronic coupling and charge-transfer energetics and may therefore represent different evolutionary solutions for achieving efficient photochemical charge separation.

Despite significant advances in multidimensional spectroscopy and first-principles calculations, several critical questions remain. For HbRC, the precise charge distribution of the initial radical pair—specifically whether the hole is delocalized across the $P_{800}\mathrm{Acc}$ region or localized as an ${\mathrm{Acc}}^{+}$ state—remains to be resolved experimentally. For PbRC, while coherent electronic and vibrational dynamics are now recognized as intrinsic features of its excitonic landscape, the field must still conclusively determine the extent to which this coherence actively enhances charge-separation efficiency rather than existing merely as a byproduct of tight pigment packing.

Moving forward, resolving these hidden vibronic signatures and overlapping transient states will require pushing the boundaries of both time-resolved spectroscopy and structural biology. The integration of time-resolved X-ray approaches, including XFEL-based measurements, with high-resolution structural information from cryo-electron microscopy (cryo-EM) could provide complementary insight into the structural coordinates associated with short-lived charge-transfer intermediates. Coupled with the continued refinement of broadband multidimensional electronic spectroscopy and advanced nonadiabatic quantum dynamics simulations, these complementary tools will be essential for disentangling the complex interplay of electronic and nuclear degrees of freedom. Ultimately, a unified spatiotemporal picture of primary photochemistry in PbRC and HbRC could deepen our understanding of photosynthetic evolution and provide useful design principles for artificial light-harvesting and solar-energy-conversion systems.

## Data Availability

No new data were created or analyzed in this study.
